# Exploring Selenoprotein P in Liver Cancer: Advanced Statistical Analysis and Machine Learning Approaches

**DOI:** 10.3390/cancers16132382

**Published:** 2024-06-28

**Authors:** Ali Razaghi, Mikael Björnstedt

**Affiliations:** Department of Laboratory Medicine, Division of Pathology, Karolinska Institutet, Karolinska University Hospital, SE-141 86 Stockholm, Sweden; mikael.bjornstedt@ki.se

**Keywords:** biomarker, hepatocellular carcinoma, hypoxia, liver cancer, machine learning, prognostic, Python, regression analysis, R programming, selenoprotein P

## Abstract

**Simple Summary:**

This research explores the role of selenoprotein P, a protein crucial for transporting selenium in the body, in liver cancer. The study aims to understand how selenoprotein P levels relate to the severity of hepatocellular carcinoma and its impact on patient outcomes. Findings indicate that selenoprotein P expression varies significantly with cancer stage and patient demographics like race and gender. It also correlates strongly with hormone and lipid metabolism markers. Importantly, selenoprotein P shows potential as a predictor of patient survival and as a biomarker for hypoxia, a condition affecting cancer progression. These insights may lead to better diagnostic tools and personalized treatments for liver cancer, emphasizing the need for further studies to validate selenoprotein P’s clinical utility in real-world settings.

**Abstract:**

Selenoprotein P (SELENOP) acts as a crucial mediator, distributing selenium from the liver to other tissues within the body. Despite its established role in selenium metabolism, the specific functions of SELENOP in the development of liver cancer remain enigmatic. This study aims to unravel SELENOP’s associations in hepatocellular carcinoma (HCC) by scrutinizing its expression in correlation with disease characteristics and investigating links to hormonal and lipid/triglyceride metabolism biomarkers as well as its potential as a prognosticator for overall survival and predictor of hypoxia. SELENOP mRNA expression was analyzed in 372 HCC patients sourced from The Cancer Genome Atlas (TCGA), utilizing statistical methodologies in R programming and machine learning techniques in Python. SELENOP expression significantly varied across HCC grades (*p* < 0.000001) and among racial groups (*p* = 0.0246), with lower levels in higher grades and Asian individuals, respectively. Gender significantly influenced SELENOP expression (*p* < 0.000001), with females showing lower altered expression compared to males. Notably, the Spearman correlation revealed strong positive connections of SELENOP with hormonal markers (AR, ESR1, THRB) and key lipid/triglyceride metabolism markers (PPARA, APOC3, APOA5). Regarding prognosis, SELENOP showed a significant association with overall survival (*p* = 0.0142) but explained only a limited proportion of variability (~10%). Machine learning suggested its potential as a predictive biomarker for hypoxia, explaining approximately 18.89% of the variance in hypoxia scores. Future directions include validating SELENOP’s prognostic and diagnostic value in serum for personalized HCC treatment. Large-scale prospective studies correlating serum SELENOP levels with patient outcomes are essential, along with integrating them with clinical parameters for enhanced prognostic accuracy and tailored therapeutic strategies.

## 1. Introduction

Selenoprotein P (SELENOP), an integral component of selenium metabolism synthesized and secreted by hepatocytes, has garnered escalating attention for its multifaceted roles in diverse physiological processes. It serves as a pivotal antioxidant, facilitating selenium transport from the liver to tissues and safeguarding against oxidative stress [1]. The expanding body of evidence suggests an intricate correlation between SELENOP expression and diverse aspects of health and disease. For example, modulation of SELENOP has been linked to obesity and diabetes, attributed to its association with insulin resistance and glucose metabolism [2,3]. SELENOP, central to thyroid hormone metabolism, influences the activation and regulation of thyroid hormones, while indirectly supporting thyroid function by contributing to the overall selenium supply and ensuring the proper activity of selenoproteins, including deiodinases crucial for thyroid hormone metabolism [4]. Additionally, SELENOP, with its selenium-containing compounds, could modulate sexual hormonal pathways, affecting the delicate balance of blood pressure regulation in women. Elevated SELENOP levels in females may influence blood pressure differently than in males, with estrogen’s vasodilatory effects impacting endothelial function and vascular tone [5].

Moreover, conflicting evidence, particularly in advanced liver disease, indicates lowered circulating selenium and SELENOP levels in hepatic diseases. Recent studies have unveiled connections between aberrant SELENOP levels and metabolic dysfunctions like non-alcoholic fatty liver disease [1]. For example, lower SELENOP levels have been observed in patients with definite non-alcoholic steatohepatitis [6]. Furthermore, lower circulating SELENOP levels [7] and hepatic mRNA SELENOP expression were observed in patients with cirrhosis [8]. 

Also, individuals diagnosed with hepatocellular carcinoma (HCC) exhibited decreased hepatic mRNA SELENOP expression in comparison to both those with liver cirrhosis and individuals with normal liver [8]. Another study reveals concentrations of selenium and SELENOP falling below healthy ranges in HCC patients, suggesting the plausible utility of SELENOP as a biomarker for monitoring and prognostic purposes during convalescence [9]. This underscores the significance of serum SELENOP as an established biomarker, not only for detecting selenium levels, but also for its ready detection in blood samples, making it particularly relevant in the context of liver-related conditions [10]. Thus, we postulate that SELENOP holds promise as a novel biomarker for prognosticating overall survival and predicting Ragnum hypoxia scores (a quantitative measure of hypoxia in tissues, derived from the expression levels of hypoxia-responsive genes [11]). Notwithstanding the longstanding utilization of serum alpha-fetoprotein (AFP) in HCC diagnosis and its historical inclusion in international guidelines, concerns regarding its diagnostic accuracy have prompted its exclusion from updated guidelines. However, debate persists regarding the continued utility of AFP [12]. This underscores the imperative for alternative biomarkers such as SELENOP, which may offer enhanced prognostic capabilities, thereby addressing potential limitations associated with AFP.

In addition, Hypoxia-Inducible Factor 1 Alpha (HIF1A) emerges as a pivotal marker for hypoxia across clinical and research domains. Functioning as a transcription factor, HIF1A orchestrates the expression of genes crucial for cellular adaptation to low oxygen levels. Elevated HIF1A expression in cancer frequently correlates with tumour hypoxia, exerting profound influences on tumour progression, metastasis, and resistance to therapeutic response. Immunohistochemistry and molecular assays represent standard methodologies for quantifying HIF1A levels, facilitating the assessment of tumour hypoxia [13]. Moreover, hypoxia reduces SELENOP export and alters selenium metabolism to favour the production of essential selenoproteins like glutathione peroxidase 4; low levels of SELENOP in the blood could indicate an adaptive response to hypoxic conditions. Monitoring SELENOP levels might help in assessing the extent of hypoxia and the body’s selenium status in various pathological conditions [14]. Therefore, our exploration of SELENOP as a potential biomarker for predicting Ragnum hypoxia scores aligns with the imperative to identify robust markers capable of informing therapeutic strategies and patient outcomes in the context of tumour hypoxia, thereby complementing the established role of HIF1A.

In general, this study aims to comprehensively investigate SELENOP’s diverse roles in liver cancer, guided by the rationale that its multifaceted functions warrant exploration using machine learning approaches for the discovery of novel biomarkers in HCC translational medicine [15]. Recent findings indicate SELENOP’s potential as a biomarker for monitoring hepatic diseases, including HCC, and as a prognostic indicator, potentially surpassing traditional markers like serum AFP. Additionally, exploring SELENOP’s correlation with Ragnum hypoxia scores complements HIF1A’s role and aids in identifying robust therapeutic indicators. Leveraging data from The Cancer Genome Atlas (TCGA), this study seeks to bridge existing knowledge gaps by evaluating SELENOP expression across various disease stages, grades, and racial and gender groups in liver cancer. It aims to ascertain SELENOP’s potential as both a prognostic marker for overall survival and a predictor for hypoxia. Furthermore, the investigation aims to unravel the intricate connections between SELENOP and hormonal/metabolic biomarkers, shedding light on the molecular mechanisms underlying SELENOP’s involvement in liver cancer for the development of novel diagnostic and therapeutic strategies.

## 2. Methods

The study utilized mRNA expression data from the Liver Hepatocellular Carcinoma (LIHC) collection in TCGA via cBioPortal (https://www.cbioportal.org/, accessed on 19 December 2023), encompassing 372 patients diagnosed with HCC. Genetic data were log-transformed mRNA expression z-scores, calculated relative to normal samples based on log RNA Seq V2 RSEM values to ensure a normalized and standardized representation of mRNA expression levels. The Shapiro–Wilk test assessed the normality of the “SELENOP” variable (W = 0.93522, *p*-value = 1.578 × 10^−11^), indicating a significant departure from the normal distribution.

### 2.1. Python Programming

The methodology employed Python alongside essential libraries, including NumPy (version 1.23.5), Pandas (version 1.5.3), Matplotlib (version 3.7.0), Seaborn (version 0.12.2), and Statsmodels (version 0.13.5). The dataset, stored as an Excel file, was initially imported using Pandas for comprehensive data manipulation and analysis. Preprocessing steps, such as converting non-numeric values to NaN and subsequently removing corresponding rows with missing data, were conducted. Statistical analysis involved Scipy’s Kruskal–Wallis test to evaluate differences among groups, generating a test statistic and a *p*-value, with a low *p*-value indicating significant differences between groups (α = 0.05). Additionally, post hoc tests, such as Dunn’s test or pairwise Mann–Whitney U tests, were applied to conduct pairwise comparisons between groups, elucidating specific group differences in “SELENOP” levels across different categories such as grade, race, category, and stage. The relationship between “SELENOP” and a derived “Category” variable, where categorization was based on “Sex” and a −0.5 to 0.5 “SELENOP” threshold of log RNA expression, was analyzed. Visualizations were addressed through Seaborn and Matplotlib, with Seaborn’s boxplot functions employed to visualize the distribution of “SELENOP” values across distinct categories. Jittered points were overlaid on the boxplots to enhance data visibility. The resulting multipanel boxplots were optimized for publication standards using Matplotlib.

The analysis also focused on exploring relationships between variables (SELENOP, Ragnum Hypoxia Score and Overall Survival) through regression modelling using Generalized Linear Models (GLM) from the Statsmodels library, specifically employing Gaussian family functions. Visualizations were crafted with Seaborn and Matplotlib, with each plot annotated to exhibit statistical metrics like *p*-values, Bayesian Information Criterion (BIC), Akaike Information Criterion (AIC), and pseudo-R-squared values. Subsequently, the analysis transitioned towards predictive modelling utilizing machine learning techniques. Although the provided code snippet lacks explicit implementation of machine learning algorithms such as Support Vector Regression or Random Forest Regression, it is essential to specify and train these models using relevant features like “SELENOP”, “Ragnum Hypoxia Score”, and “HIF1A” to predict outcomes such as “Overall Survival” or “Ragnum Hypoxia Score”. To ensure the accuracy and effectiveness of the analysis, it is crucial to compute evaluation metrics like Mean Squared Error (MSE), R-squared, or cross-validated performance to assess the predictive accuracy of the models.

### 2.2. R Programming

Correlation and multiple regression analyses were conducted using the R programming language, version 4.3.1. Considering the non-normal distribution, subsequent statistical tests utilized non-parametric methods to ensure robustness in the analysis. To maintain analysis integrity, missing values and non-numeric data were systematically omitted from the dataset, mitigating potential biases and inaccuracies. Spearman correlations among selected variables that exhibited highly significant concurrence (“SELENOP”, “androgen receptor (AR)”, “Estrogen Receptor 1 (ESR1)”, “Thyroid hormone receptor β (THRB)”, “peroxisome proliferator-activated receptor α (PPARA)”, “Apolipoprotein C3 (APOC3)” and “Apolipoprotein 5 (APOA5)”) were conducted using R with the “Spearman” method from the psych package. The correlation matrix was printed, and a scatterplot matrix with correlation values was generated using the same package.

A conditional check verified sufficient data points for linear regression analysis. If met, a multiple linear regression model was created using the lm function from the stats package, regressing “SELENOP” on “ESR1”, “THRB”, “AR”, and “APOC3”.

## 3. Results

### 3.1. SELENOP Expression by Cancer Grade

**Figure 1:**
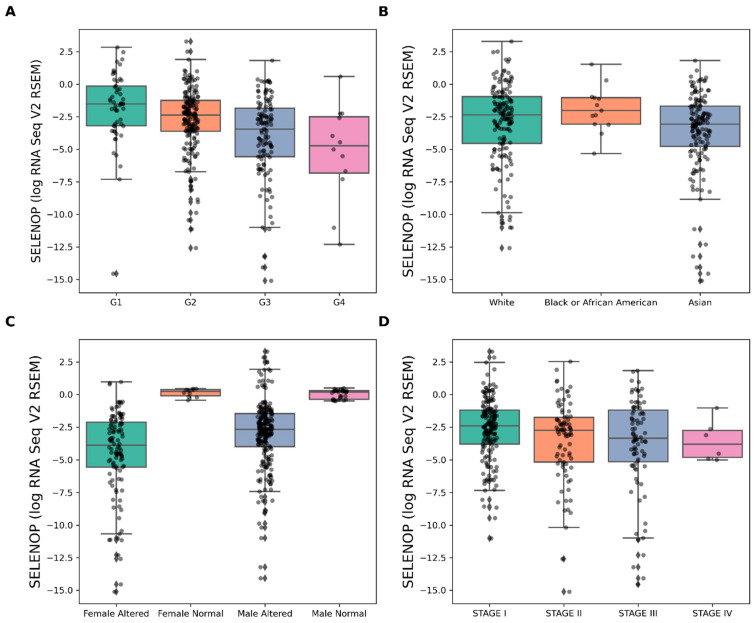
SELENOP expression in different subpopulations of patients diagnosed with HCC. (**A**) based on the histological grade of cancer (G1: well-differentiated tumors or low grade, G2: moderately differentiated or intermediate grade, G3: poorly differentiated or high grade, G4: undifferentiated or highest grade). (**B**) based on racial groups in the USA. (**C**) based on gender. (**D**) based on the Neoplasm Disease Stage American Joint Committee on Cancer Code (Stage I: denotes a small-sized tumor confined to the organ of origin; Stage II: indicates a disease that has locally advanced beyond the original site. Stage III: is characterized by the spread of the disease to neighbouring organs, and Stage IV: signifies the presence of distant metastatic disease).

The levels of SELENOP were assessed across four grades of liver cancer using the Kruskal–Wallis test, revealing a statistically significant difference among the groups (H-statistic = 30.16, *p* < 0.000001). Subsequent pairwise Dunn’s tests were conducted to further elucidate these differences. The comparison between Grade 1 (G1) and Grade 2 (G2) exhibited a significant discrepancy (*p* = 0.0336), as did G1 versus G3 (*p* = 0.0000123) and G1 versus G4 (*p* = 0.00983). Similarly, significant differences were observed between G2 and G3 (*p* = 0.000307) as well as G2 and G4 (*p* = 0.0428). However, the comparison between G3 and G4 did not reach statistical significance (*p* = 0.297), suggesting a similarity in SELENOP levels between these two grades of liver cancer (Figure 1A). These findings underscore the potential utility of SELENOP as a biomarker for distinguishing different grades of liver cancer.

### 3.2. SELENOP Expression across Races

The Kruskal–Wallis test revealed a significant association between SELENOP levels and race (H-statistic = 7.41, *p* = 0.0246). Pairwise Dunn’s tests identified significant differences in SELENOP levels between Asian and Black or African American individuals (*p* = 0.00692), suggesting racial disparities. While comparisons between Asian and White individuals (*p* = 0.0622), and between Black or African American and White individuals (*p* = 0.0806), did not reach significance (Figure 1B). These findings underscore racial influences on SELENOP expression levels.

### 3.3. SELENOP by Gender

The Kruskal–Wallis test indicated a highly significant association between SELENOP levels and gender (H-statistic = 87.49, *p* < 0.000001). Subsequent pairwise Dunn’s tests unveiled significant differences in SELENOP expression across different gender groups. Specifically, significant distinctions were observed between Male Altered and Male Normal individuals (*p* < 0.000001), Male Altered and Female Altered individuals (*p* = 0.000123), Male Altered and Female Normal individuals (*p* < 0.000001), Male Normal and Female Altered individuals (*p* < 0.000001), and Female Altered and Female Normal individuals (*p* < 0.000001). Notably, female-altered individuals exhibited the lowest SELENOP expression levels. However, no statistically significant difference was found between Male Normal and Female Normal individuals (*p* = 0.289) (Figure 1C). These results highlight distinct SELENOP expression patterns across gender and altered states, with the noteworthy observation that Female Altered is also lower than Male Altered, providing valuable insights into potential biological and hormonal variations.

### 3.4. SELENOP by Stage of Cancer

The Kruskal–Wallis test revealed no statistically significant association between SELENOP levels and stage of cancer (H-statistic = 6.84, *p* = 0.0771), suggesting that SELENOP levels are consistent among the patients with the four stages of HCC (Figure 1D).

### 3.5. SELENOP as a Prognosticator of Overall Survival

**Figure 2:**
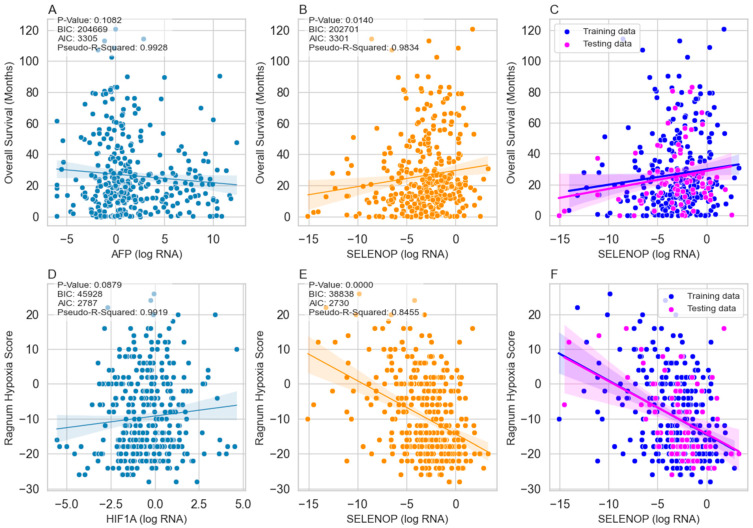
Relationships between biomarkers and Ragnum Hypoxia Score in HCC patients. (**A**) displays Overall Survival versus AFP serving as standard clinical control. (**B**) shows Overall Survival versus SELENOP. (**C**) illustrates Overall Survival predicted by a machine learning model based on SELENOP levels, with blue and magenta points representing training and testing data, respectively. (**D**) depicts Ragnum Hypoxia Score versus HIF1A, serving as standard clinical control. (**E**) exhibits Ragnum Hypoxia Score versus SELENOP. (**F**) shows the Ragnum Hypoxia Score predicted by a machine learning model based on SELENOP levels, with blue and magenta points representing training and testing data, respectively. Solid lines indicate the fitted regression lines. Abbreviations: AFP (Alpha-fetoprotein), AIC (Akaike Information Criterion), BIC (Bayesian Information Criterion), HIF1A (Hypoxia-inducible factor 1 alpha).

Our investigation employed a generalized linear model (GLM) due to the non-normal distribution of SELENOP expression levels. Despite this non-normality, the GLM allowed us to effectively analyze the association between SELENOP expression and Overall Survival in HCC patients, contrasting its performance with that of the established AFP biomarker. Regression analysis yielded a statistically significant association between SELENOP expression levels and Overall Survival (*p*-value = 0.014), while for AFP, the *p*-value was 0.1082. The BIC and AIC values for the SELENOP model were 202,701 and 3301, respectively, whereas for AFP, these values were 204,669 and 3305. These results suggest a superior model fit for SELENOP compared to AFP. Additionally, the pseudo-R-squared value of 0.9834 for the SELENOP model indicates a high degree of explained variance in Overall Survival, while for AFP, the pseudo-R-squared value was 0.9928 (Figure 2A,B). Despite AFP being a commonly utilized biomarker in clinical settings, our analysis revealed a weaker association with OS compared to SELENOP. These findings underscore the potential utility of SELENOP as a prognostic biomarker in HCC patients.

Subsequent to our regression analysis, we further evaluated the predictive performance of SELENOP expression levels for Overall Survival in HCC patients using machine learning techniques. The prediction results revealed a mean squared error of 409.610 and an R-squared value of 0.0285. These metrics suggest that while SELENOP expression levels provide some predictive value regarding patient outcomes, the model’s performance is limited, with only a small proportion of the variance in outcomes explained by SELENOP alone (Figure 2C).

### 3.6. SELENOP as a Predictor of Hypoxia

We utilized a GLM regression approach to evaluate the efficacy of SELENOP expression as a biomarker for predicting hypoxia in HCC patients, contrasting its performance with that of the established HIF1A biomarker. Our findings revealed compelling evidence supporting the predictive potential of SELENOP. Notably, the GLM regression model for SELENOP exhibited a highly significant *p*-value of 0.0, indicative of a robust association with the Ragnum Hypoxia Score. Moreover, the BIC and AIC values for the SELENOP model were substantially lower than those for HIF1A, suggesting a superior model fit for SELENOP. The pseudo-R-squared value of 0.8455 further reinforces the efficacy of SELENOP in explaining variance in hypoxia scores. Conversely, while HIF1A also did not demonstrate a significant association with the Ragnum Hypoxia Score (*p*-value = 0.0879), its model fit, as indicated by BIC and AIC values, was inferior to that of SELENOP. Nonetheless, the pseudo-R-squared value for HIF1A remained relatively high at 0.9919 (Figure 2D,E). These findings underscore the potential of SELENOP as a promising biomarker for predicting hypoxia in HCC patients, potentially rivalling, or surpassing the predictive power of HIF1A.

Following our GLM regression analysis, we extended our investigation to assess the predictive performance of SELENOP expression for hypoxia in HCC patients using a machine-learning approach. The prediction results demonstrated a mean squared error (96.68) and an R-squared value (0.1889). These findings suggest that while SELENOP expression levels exhibit some predictive capability for hypoxia, the model’s performance remains moderate, with approximately 18.89% of the variance in hypoxia scores explained by SELENOP alone (Figure 2F).

The predictive Equation (1) establishes the link between SELENOP (log RNA) levels and the Ragnum Hypoxia Score. Despite the moderate predictive performance, the machine learning model highlights SELENOP’s potential utility as a predictive biomarker for hypoxia in HCC patients; it offers valuable insight into estimating hypoxia severity in HCC patients.

Ragnum Hypoxia Score = −14.4486 − 1.5353 × SELENOP (log RNA)(1)

### 3.7. Spearman Correlation Matrix Reveals Complex Associations of SELENOP with Hormonal and Metabolic Biomarkers

**Figure 3:**
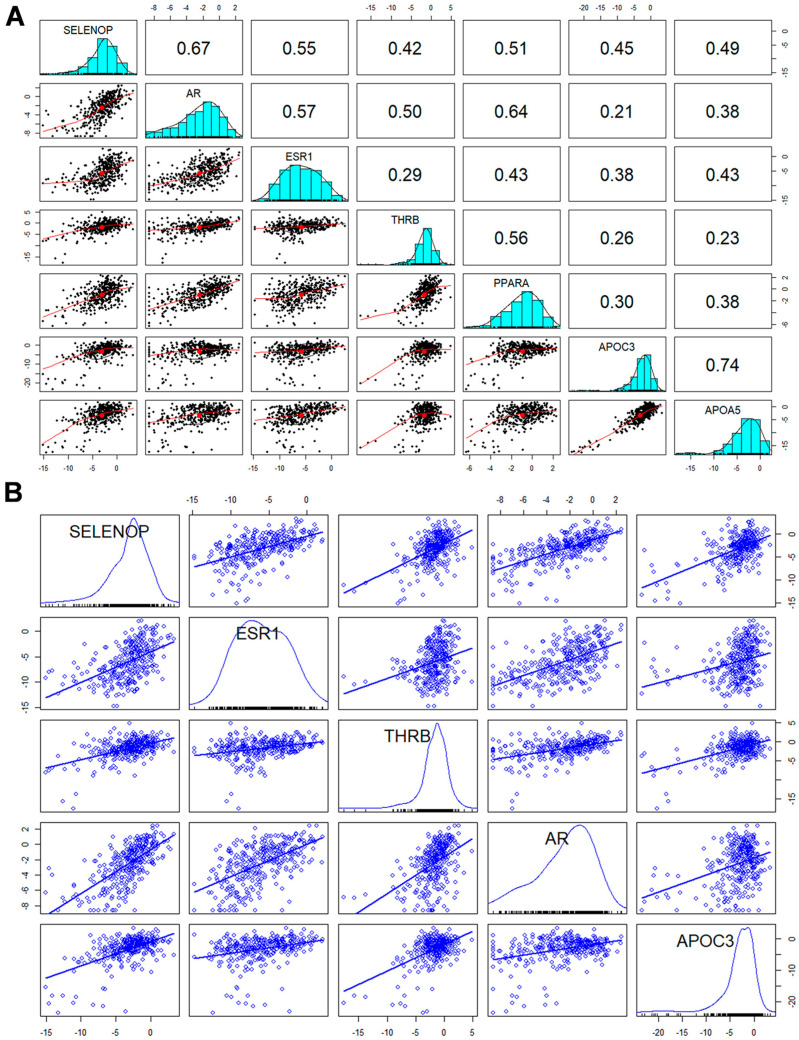
(**A**) Spearman correlation matrix and scatterplot matrix (1 > ρ ≥ 0.8: Strong positive correlation; 0.8 > ρ ≥ 0.4: Moderate positive correlation, 0.4 > ρ > 0: Weak positive correlation). The Spearman correlation matrix displays the strength and direction of relationships between SELENOP and key variables (AR, ESR1, THRB, PPARA, APOC3, APOA5). In this context, the correlation between SELENOP and AR is notably strong (0.6681), suggesting a robust positive correlation. The relationship between SELENOP and ESR1 is of moderate-to-strong magnitude (0.5470), indicating a substantial connection. A moderate positive correlation is observed with APOC3 (0.4480). The scatterplot matrix complements these findings, providing visual insights into the paired variable relationships. (**B**) Scatterplot matrix and multiple regression model. The figure showcases a scatterplot matrix elucidating relationships between SELENOP and the variables ESR1, THRB, AR, and APOC3, with blue lines indicating linear regression planes. Employing a multiple regression model, SELENOP was modelled as a function of ESR1, THRB, AR, and APOC3. Notable coefficients include ESR1 (Est. = 0.13069, *p* = 0.000516), THRB (Est. = 0.12307, *p* = 0.019482), AR (Est. = 0.48668, *p* < 2 × 10^−16^), and APOC3 (Est. = 0.23784, *p* = 1.73 × 10^−13^). The model’s strong fit is evidenced by a residual standard error of 1.905 on 327 degrees of freedom and an R-squared value of 0.5957. The mRNA expression levels analyzed in this study were derived from cancerous tissue samples of patients diagnosed with HCC. Abbreviations: APOA5—Apolipoprotein 5, APOC3—Apolipoprotein C3, AR—Androgen Receptor, ESR1—Estrogen Receptor 1, PPARA—Peroxisome Proliferator-Activated Receptor α, SELENOP—Selenoprotein P.

The intricate web of relationships between SELENOP and a set of pertinent biomarkers, each representing distinct physiological domains, was investigated. Notably, SELENOP exhibited strong positive correlations with biomarkers associated with hormonal changes, such as AR (ρ = 0.668), ESR1 (ρ = 0.547), and THRB (ρ = 0.424). These findings underscore the potential involvement of SELENOP in the intricate regulation of hormonal dynamics. Furthermore, our analysis revealed compelling connections between SELENOP and biomarkers implicated in lipid/triglyceride metabolism, including strong-to-moderate positive correlations with PPARA (ρ = 0.507), APOC3 (ρ = 0.448), and APOA5 (ρ = 0.485). These associations highlight the potential role of SELENOP in influencing lipid homeostasis. Moreover, the correlations with THRB (ρ = 0.424) and APOC3 (ρ = 0.448) reveal intricate details in the interrelationships between thyroid hormones and lipid metabolism in HCC, prompting additional exploration to decipher the molecular mechanisms underlying these connections (Figure 3A).

### 3.8. Robust Regression Analysis Reveals Hormonal and Metabolic Influences on SELENOP mRNA Expression in Liver Cancer

The multiple linear regression model, examining the relationship between SELENOP mRNA expression and predictor variables (ESR1, THRB, AR, and APOC3), demonstrated a high level of fitness. The model’s overall significance (the *p*-value = 2.2 × 10^−16^), affirms its robustness. The coefficients for each predictor variable suggest their significant individual contributions to SELENOP expression. Specifically, elevated levels of ESR1, THRB, AR, and APOC3 correlate with increased SELENOP mRNA expression. While PPARA and APOA5 are correlated to SELENOP, these two biomarkers do not significantly affect SELENOP in the regression analysis and were therefore omitted from the final model to improve model parsimony and avoid multicollinearity. The model’s goodness of fit was further emphasized by the F-statistic, with a high value of 120.5 on 4 and 327 degrees of freedom, highlighting its statistical strength. The R-squared and adjusted R-squared values of 0.5957 and 0.5908, respectively, signify that approximately 60% of the variability in SELENOP expression is accounted for by the included predictors. This attests to our model’s high efficacy in capturing and explaining the observed patterns in SELENOP mRNA expression within the context of liver cancer (Figure 3B).

## 4. Discussion

In summary, SELENOP displays nuanced relationships across diverse disease stages and grades, racial and gender groups, and overall survival. Noteworthy distinctions in SELENOP levels among specific subgroups, including varying cancer grades and different racial or gender categories, accentuate the complexity of its involvement in liver cancer. These findings imply a potentially distinct role for SELENOP in diverse HCC subpopulations. Notably, SELENOP levels were lower in high-grade (G3–G4) compared to low-grade (G1) cancers, suggesting a correlation with cancer severity. Racial disparities were observed, with individuals of Asian descent exhibiting lower SELENOP expression than Black or African American individuals. Gender-specific variations were evident, with female patients, particularly in altered states, displaying lower SELENOP levels than males. The Spearman correlation matrix illuminates compelling connections between SELENOP and biomarkers involved in both hormonal dynamics and lipid metabolism. Robust positive correlations are evident with key hormonal markers such as AR (ρ = 0.668), ESR1 (ρ = 0.547), and THRB (ρ = 0.424), underscoring the intricate involvement of SELENOP in hormonal pathways within the realm of liver cancer. Additionally, the matrix reveals strong-to-moderate positive correlations between SELENOP and biomarkers implicated in lipid metabolism, further enriching our understanding. Noteworthy associations include those with PPARA (ρ = 0.507), APOC3 (ρ = 0.448), and APOA5 (ρ = 0.485). Furthermore, the subsequent robust regression model not only corroborates these intricate correlations but accentuates the substantial impact of both hormonal and lipid metabolic factors on SELENOP mRNA expression in the context of liver cancer. The model’s high significance, well-defined coefficients, and strong goodness-of-fit metrics collectively underscore the complexity and efficacy of the associations observed in the Spearman correlation matrix.

In addition, through advanced statistical analyses and machine learning techniques, we have demonstrated a statistically significant association between SELENOP expression levels and overall survival rates among HCC patients, highlighting its potential as a prognostic indicator. However, our investigation also reveals the complexity of SELENOP’s predictive capabilities, with the machine learning model for overall survival exhibiting limited performance; this underscores the complexity of HCC prognosis, which likely involves multifactorial interactions beyond SELENOP expression alone. Consequently, while SELENOP shows promise as a prognostic biomarker, its predictive utility may be enhanced when integrated with other clinical, pathological, and molecular factors.

In contrast, the model for hypoxia demonstrated moderate efficacy, shedding light on SELENOP’s predictive potential for hypoxia in HCC, and offering valuable insights into its role in tumour microenvironment dynamics.

### 4.1. Biological Plausibility and Mechanistic Insights

The multifaceted nature of SELENOP in cancer encompasses both selenium transport and antioxidant functions. Unlike most selenoproteins, SELENOP, primarily produced in the liver, is known for its roles in selenium transport. Incorporating selenium into up to 10 selenocysteine residues, SELENOP is secreted into plasma, reaching distant tissues before lysosomal degradation, and contributing to the synthesis of other selenoproteins (e.g., GPXs) [16]. While SELENOP is often a reliable marker for overall selenium levels [17], epidemiological studies reveal varied correlations with cancer, often with decreased levels observed in several tumour types, including hepatobiliary cancer [17]. Our analysis also confirms the same trend in HCC as well.

The central role of SELENOP in regulating thyroid hormones [4] and sex-specific hormones like estrogen have already been discussed [5]. In our study, the strong positive correlations with biomarkers associated with hormonal changes, such as AR, ESR1, and THRB, suggest a role in estrogenic and thyroid dynamics.

In the context of lipid metabolism, several inconsistent reports show either negative or positive correlations between SELENOP and lipoproteins (e.g., HDL and LDL) [18]. SELENOP engages in cellular uptake through apoER2 and megalin receptors, both part of the lipoprotein receptor family. ApoER2 mediates endocytosis in the brain and testicles, while megalin serves as the primary receptor in the kidneys. These receptors, with additional functions impacting tumour growth, highlight the complexity of SELENOP’s role. ApoER2 regulates cell motility, and increased megalin expression influences tumour cell proliferation [16]. The observed correlation of reduced SELENOP levels with fatty liver disease has also been documented [1,6].

APOA5, exclusively synthesized by the liver, plays a vital role in triglyceride metabolism, being associated with chylomicrons, LDL, and HDL in plasma [19]. APOC3 is also a widely known key player in triglyceride metabolism [20]. PPARA similarly plays a vital role in lipid metabolism [21]. To our knowledge, our study is the inaugural report establishing a robust correlation and association between SELENOP and markers of lipid and triglyceride metabolism in HCC. The identified connections with key lipid/triglyceride metabolism biomarkers, namely PPARA, APOC3, and APOA5, suggest the involvement of SELENOP in lipid homeostasis within the context of liver cancer.

### 4.2. Clinical Implications

The identified associations between SELENOP and various clinical parameters in liver cancer underscore its potential as a biomarker for diagnostic, prognostic, and therapeutic purposes [9]. Furthermore, the detectability of SELENOP in serum and/or plasma underscores its potential as a non-invasive liquid biopsy marker, presenting clinicians with a minimally intrusive avenue for diagnostic insights [10]. The consistent expression profile of SELENOP across distinct stages of HCC, as revealed by this analysis, suggests its promise as a diagnostic biomarker, particularly in the early stages of the disease. The stability of SELENOP levels across disease stages, coupled with its presence in easily accessible biofluids, positions SELENOP as a prospective asset in advancing early detection strategies for HCC [22]. More importantly, SELENOP shows promise as a biomarker for predicting hypoxia, potentially rivalling established markers such as HIF1A.

In addition, based on our results, monitoring SELENOP levels alongside key biomarkers, including hormonal markers (AR, ESR1, THRB) and those associated with lipid/triglyceride metabolism (PPARA, APOC3, APOA5), may offer a comprehensive approach for refining risk stratification and guiding personalized treatment strategies in liver cancer. Regular assessment of SELENOP, in conjunction with these relevant biomarkers, holds promise for improving the effectiveness of personalized treatment approaches and ultimately enhancing patient outcomes.

Integrating SELENOP into routine clinical practice could revolutionize liver cancer diagnostics and prognosis. As a liquid biopsy marker, SELENOP enables non-invasive monitoring of disease status and treatment response, facilitating timely, tailored interventions that improve outcomes and reduce costs.

Additionally, SELENOP’s role in predicting hypoxia could enhance HCC management by identifying patients at higher risk, allowing for early, targeted therapies that mitigate hypoxia’s adverse effects, and improving survival and quality of life.

Moreover, combining SELENOP with existing biomarkers could create a comprehensive diagnostic tool, enhancing prognostication and personalized treatment. Future research should validate these findings in diverse populations and explore SELENOP’s clinical applications.

### 4.3. Limitations

Some limitations should be considered when interpreting the results of this study. First, the cross-sectional nature of TCGA data limits the establishment of causal relationships between SELENOP and various factors in liver cancer [23]. Longitudinal genetic analysis unveils changes in disease phenotypes over time, refining disease status definitions. Repeat measurements enhance understanding of disease progression, capturing time-varying covariates and interactions often overlooked in cross-sectional studies focused on a single time point [24].

Additionally, the reliance on mRNA expression data from TCGA introduces inherent limitations, such as potential variations in sample collection methods and platforms [23]. Also, the dataset’s retrospective nature lacks specific details on lifestyle factors and comorbidities like hepatitis and cirrhosis that could influence SELENOP expression and impact the result as confounders.

### 4.4. Future Directions

In future, investigating SELENOP’s dynamic interactions with hormonal and lipid/triglyceride metabolism biomarkers is vital for nuanced comprehension of its mechanistic involvement in liver cancer. Scrutinizing correlations with specific molecular entities relevant to hormonal dynamics (e.g., AR, ESR1, THRB) and lipid/triglyceride metabolism (e.g., PPARA, APOC3, APOA5) holds promise for elucidating underlying molecular pathways. Integrating multi-omics data and advanced analytical methodologies will provide a comprehensive insight into SELENOP’s associations within these pathways, potentially identifying therapeutic targets for HCC and refining personalized treatment approaches.

To bolster the prognostic value of SELENOP for overall survival and its diagnostic utility for hypoxia in HCC, validating its expression in serum is paramount. For instance, in a multicentered study, SELENOP was inversely associated with all-cause mortality following breast cancer diagnosis [25]. Thus, further large-scale prospective studies correlating serum SELENOP levels with patient outcomes, including overall survival, are essential, particularly in liver cancer. Longitudinal analyses can track SELENOP variations over time and their association with disease progression. Integration of serum SELENOP levels with clinical parameters can enhance prognostic accuracy. Additionally, validating SELENOP as a diagnostic marker for hypoxia through correlation with imaging and hypoxia biomarkers is crucial. Prospective trials assessing SELENOP-guided treatment strategies’ impact on patient outcomes are necessary.

## 5. Conclusions

Our comprehensive study provides a thorough examination of SELENOP’s involvement in HCC, revealing its diverse associations across disease stages and grades, racial and gender groups, and overall survival outcomes. Significant variations in SELENOP expression levels were observed among specific subgroups, particularly across different cancer grades and demographic categories, highlighting its nuanced role in HCC pathogenesis. Lower SELENOP levels in high-grade cancers suggest a potential correlation with disease severity, while disparities across racial and gender groups underscore the complexity of SELENOP’s influence in diverse patient populations. Additionally, robust regression analyses uncover connections between SELENOP and biomarkers involved in hormonal dynamics and lipid metabolism, shedding light on its mechanisms of action in HCC progression. Notably, our study identifies SELENOP as a potential prognostic biomarker, with significant associations observed with overall survival rates among HCC patients. While its predictive capabilities are moderately limited, SELENOP shows promise as a biomarker for predicting hypoxia, potentially rivalling established markers such as HIF1A. Nonetheless, it is essential to acknowledge the limitations of our study, including the cross-sectional nature of TCGA data and potential biases inherent in mRNA expression analyses. Future research should focus on validating SELENOP’s prognostic and diagnostic utility in serum through large-scale prospective studies while integrating multi-omics data to elucidate its intricate molecular pathways in HCC.

## Data Availability

The data analyzed in this study were obtained from The Cancer Genome Atlas (TCGA) database, which is publicly available at https://www.cancer.gov/tcga, accessed on 19 December 2023. Researchers can access the data through the Genomic Data Commons Data Portal following TCGA’s data access policies. Any additional data supporting the findings of this study are available from the corresponding author upon reasonable request.

## Abbreviations

AFP—Alpha-fetoprotein, AIC—Akaike Information Criterion, APOA5—Apolipoprotein 5, APOC3—Apolipoprotein C3, AR—Androgen Receptor, BIC—Bayesian Information Criterion, ESR1—Estrogen Receptor 1, GLM—Generalized Linear Model, HIF1A—Hypoxia-Inducible Factor 1 Alpha, LIHC—Liver Hepatocellular Carcinoma, MSE—Mean Squared Error, PPARA—Peroxisome Proliferator-Activated Receptor α, R-squared—Coefficient of Determination, SELENOP—Selenoprotein P, TCGA—The Cancer Genome Atlas, THRB—Thyroid Hormone Receptor β.
